# Supplementary material for: SMRT Sequencing Enables High-Throughput Identification of Novel AAVs from Capsid Shuffling and Directed Evolution
Source: Genes (Basel). 2023 Aug 21;14(8):1660. doi: 10.3390/genes14081660 (PMC10454592; doi:10.3390/genes14081660)
Supplement: Supplementary file 1 [file genes-14-01660-s001.zip › genes-2544982-supplementary.pdf]

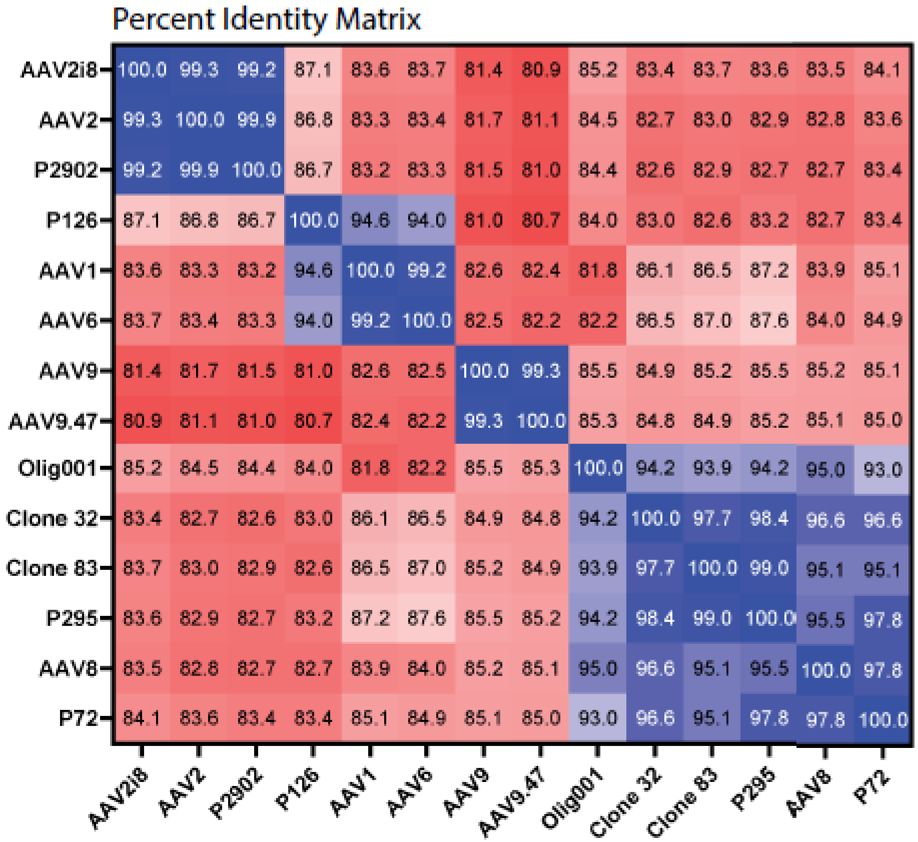


**Figure S1. Percent identity matrix of parent and select lead candidates.** Heatmap shows a percent identity after pairwise sequence alignment of each parent and lead candidate capsid.
